# Differential co-occurrence analysis: a method to extract ecological modules from clinical microbiome data

**DOI:** 10.1128/msystems.00284-26

**Published:** 2026-06-09

**Authors:** J. Iacovacci, N. Cannon, J. A. McCulloch, T. Rancati, G. Trinchieri

**Affiliations:** 1 Data Science Unit, Fondazione IRCCS Istituto Nazionale dei Tumori di Milano 9329, Milan, Italy; 2 Laboratory of Integrative Cancer Immunology, National Cancer Institute Center for Cancer Research 272101, Bethesda, Maryland, USA; Florida Atlantic University, Boca Raton, Florida, USA

**Keywords:** microbiome data, ecological networks

## Abstract

The human microbiota plays a pivotal role in health, with widespread alterations implicated in conditions ranging from inflammatory disorders to cancer. While correlation-based network analyses have illuminated ecological interactions within these communities, the host environment uniquely mediates microbial relationships, demanding new methods to capture dynamic, condition-dependent modules of species interactions. Here, we present a statistical framework termed differential co-occurrence analysis, which identifies blocks of taxa whose collective presence is strengthened or weakened under distinct host states. By leveraging recent advances in metagenomics that enable detailed taxonomic profiling and higher-order interaction discovery, our method transcends traditional pairwise correlation constraints. Conceptually akin to associative rule mining, it diverges through the integration of robust statistical modeling, directly extracting interactions that differ significantly between conditions. This approach offers a refined lens to dissect microbiota ecology and could pave the way for new insights into microbiome-associated disease mechanisms.

**IMPORTANCE:**

The research on the role of the intestinal microbiota in the onset of cancer and as a modulator of anticancer treatments, including chemotherapeutics and immune checkpoint inhibitors, is helping medicine to identify novel strategies for cancer prevention, for the delivery of more effective treatments, and in reducing treatment side effects and complications. Within this context, it is of crucial importance to approach the analysis of clinical microbiome data with an ecology-oriented perspective and to develop bioinformatics tools able to identify functional interactions in bacterial communities of patients from observational cohort studies. Clinical microbiome datasets are typically high dimensional, comprising numerous taxa measured across relatively few samples. This imbalance increases the risk of statistical overfitting and undermines the robustness of analytical findings. However, recent advances in metagenomic bioinformatics pipelines and reference databases have enabled the comprehensive extraction of genetic information from microbiome samples, facilitating the precise characterization of bacterial species presence and absence. In our manuscript, we describe a statistical computational method that we named differential co-occurrence analysis, which focuses on the analysis of the co-presence of microbiota taxa across samples associated with different host conditions. The proposed method can reveal modules of interacting taxa that are strengthened or weakened when the host condition changes (e.g., when passing from a healthy state to a disease state). The method is general and applicable to a broad range of ecological datasets featuring presence/absence data structures. Furthermore, the method accommodates the analysis of higher-order co-occurrence patterns beyond pairwise co-occurrence, thereby enabling the investigation of higher-order interactions, whose detection and identification are a major challenge in ecological network analysis.

## INTRODUCTION

The microbiota, a term which describes the populations of microorganisms living on the surfaces (exposed or not) of host animals and plants, including humans, is now recognized to be an essential factor for health, with microbiota alterations almost universally observed and often proved to be directly involved in most human disease conditions, ranging from inflammatory states to autoimmune diseases and cancer (1–4). To advance medicine, it is therefore essential to understand the ecology of microbiota systems and their interaction with the host, which ultimately represents their natural environment (3, 5, 6).

Correlation-based techniques developed in the field of ecology have been commonly used to extract ecological networks from microbiome data mainly through the analysis of changes in relative abundances of species between host conditions (7–9). However, correlations in abundances between species in the microbiota are always mediated by the host environment differently from animal and plant scale ecosystems (10, 11). For example, within the human gut environment, different mechanisms contribute to control the stability and abundance of species in the gut microbiota community under healthy conditions, including immune system-mediated regulation (5).

On the other hand, modularity (i.e., the design of a system as an ensemble of separate, independent modules or components) is considered a universal property of biological systems and of ecosystems (12, 13). Recent studies have provided evidence in support of the modular nature of the microbiota interaction network, revealing the coexistence of different network modules associated with different metabolic functions (14–16).

Here, we propose a computational method named differential co-occurrence analysis that can be used to extract blocks (modules) of interactions between taxa from microbiome data that are strengthened or weakened when host condition changes between two states, for example, passing from a healthy state to a specific disease condition.

The method is based on the analysis of the co-presence of microbiota taxa across samples associated with different host conditions. Furthermore, co-presence is general with respect to the extraction of microbial interactions beyond pair-wise, often called higher-order interactions (HOIs), whose detection and identification are a major challenge in microbial network inference (17).

The expansion of taxonomy reference databases and the advances in microbial genome reconstruction enable an exhaustive extraction of genetic information from microbiome samples, resulting in reliable data sets on the presence/absence of specific bacterial species and strains (18–20).

The proposed algorithm is conceptually related to the Apriori algorithm (and, in general, with associative rule mining techniques) that also places the focus on the analysis of co-presence to infer interactions and that have been recently applied to microbiome data (11, 21, 22). However, two fundamental aspects of the algorithm make the proposed methodology novel and suitable for the analysis of microbiota ecological interactions in different host conditions. First, our method enforces the construction of sophisticated statistical models directly from the data, thus avoiding the use of simple frequency thresholds for statistical testing of interactions.

Second, instead of extracting multiple condition-dependent networks and comparing them, the method is designed to directly extract networks that are statistically dissimilar between conditions.

## MATERIALS AND METHODS

Given the modularity of microbial network interactions, we hypothesized that different ecosystem blocks or modules coexist within the microbiota and that changes in the host environment (e.g., disease conditions) might favor certain blocks of interacting species over others. To test this hypothesis and to detect ecosystem blocks associated with specific host conditions, we developed a methodology (with an associated computational pipeline) that we named differential co-occurrence analysis.

### Benchmark microbiome data sets

To benchmark the proposed method, we used different clinical microbiome data sets as follows.

The Pittsburgh melanoma cohort included stage III or IV melanoma cancer patients (*n* = 63) treated at the University of Pittsburgh’s Hillman Cancer Center whose gut microbiota was profiled from stool samples collected before treatment or within 4 months of starting anti-PD-1 immunotherapy (still representative of baseline given that the microbiome of patients treated with anti-PD-1 is stable early after therapy initiation) using shotgun metagenomic sequencing (23).

The Gupta CRC cohort included colorectal cancer (CRC) patients and healthy individuals (*n* = 59) recruited from two different locations (Bhopal and Kerala) in India, whose gut microbiota was profiled from stools using shotgun metagenomic sequencing (24).

The IEO CRC cohort included CRC patients and healthy controls matched by age (*n* = 60), enrolled at the European Institute of Oncology (IEO) of Milan, Italy, whose gut microbiota was profiled from stools using shotgun metagenomic sequencing (25).

### Data processing

For CRC cohorts, raw read sequences were downloaded from the NCBI database (Gupta cohort, BioProject accession numbers PRJNA531273 and PRJNA397112; IEO cohort accession number PRJNA447983) and processed using JAMS (version 1.9.2) (20). In addition to the domain, kingdom, phylum, class, order, family, genus, species, and strain (infra-species) levels, JAMS computes the last known taxon (LKT), which is the lowest non-missing, non-unclassified taxonomic level for a particular NCBI taxid. Pittsburgh cohort data are also available in the NCBI database, BioProject accession number PRJNA762360. Details on samples metadata are reported in Data S1.

### Differential co-occurrence analysis

**Fig 1:**
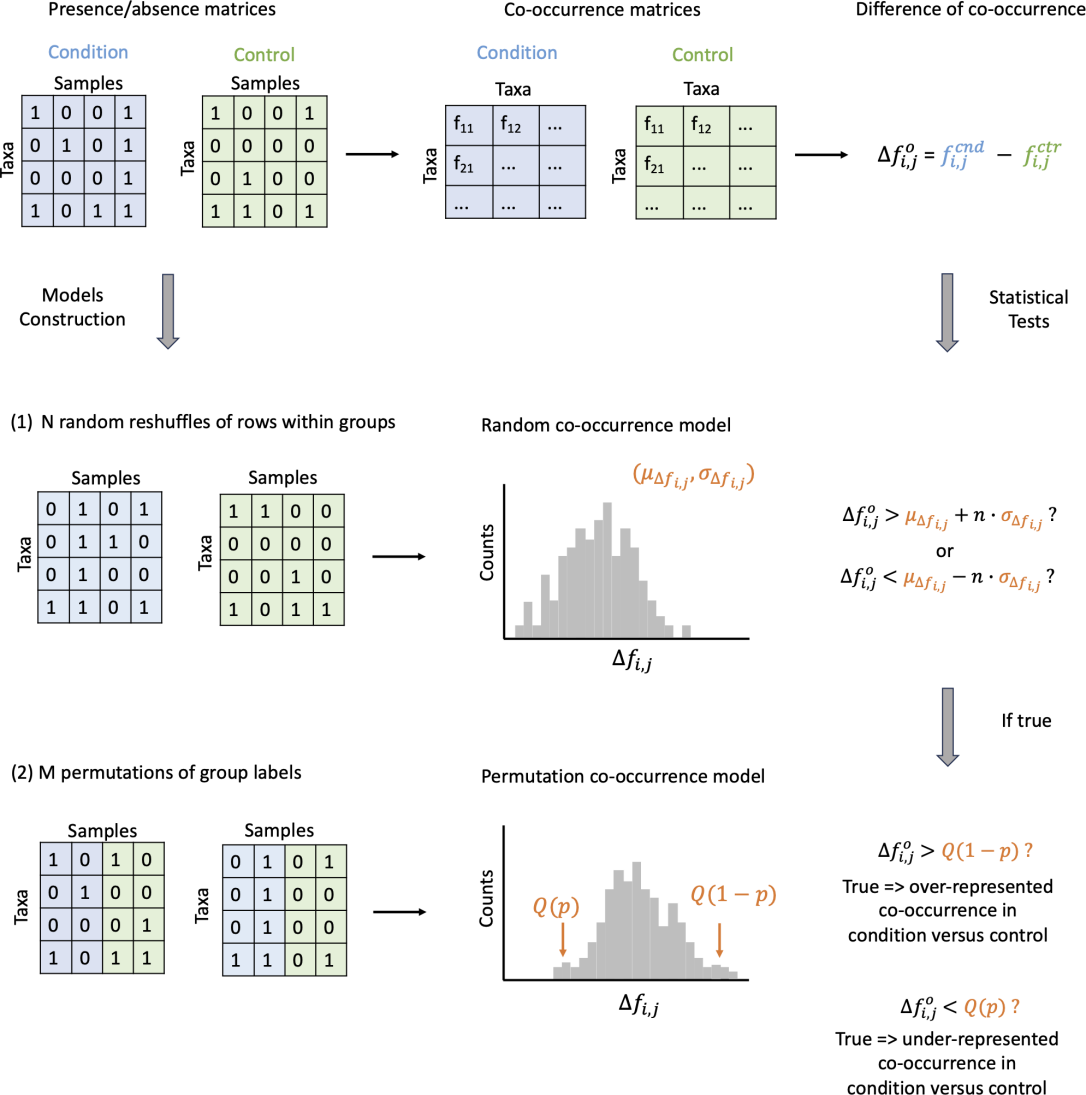
Differential co-occurrence analysis: diagram schematizing the steps of the differential co-occurrence analysis.

The differential co-occurrence analysis procedure is schematized in Fig. 1. The method can be applied in general to ecological occurrence matrices with dimensions equal to the number of taxa times the number of samples, describing presence/absence in distinct environments.

In the analysis of clinical microbiome data sets, for which the method is designed, samples often describe the relative abundance profiles of taxa and are grouped according to the host conditions of interest, e.g., samples from non-responder patients and from responder patients in the Pittsburgh melanoma cohort (“condition” versus “control” in Fig. 1).

Occurrence (presence/absence) matrices with dimension equal to the number of taxa times the number of samples are computed for the samples in the control group and in the condition group, respectively. These binary matrices recapitulate the information of taxa presence across samples in each condition, with element 1 corresponding to taxa relative abundance greater than or equal to detection threshold (default value 0.01%) or 0 otherwise.

Co-occurrence matrices with dimension equal to the number of taxa squared are then obtained by computing the frequencies at which different pairs of taxa are co-present in samples associated with the same host condition, mathematically


$$f_{i,j}^{k}=\frac{1}{n_{k}}\sum_{l\;\in \;condition\;k}O_{i,l}\cdot O_{j,l},$$

where k is the condition index, i and j are taxon indices, and l is a sample index, $n_{k}$ is the number of samples associated with condition k, and $O_{i,l}$ describe the occurrence of taxon i in sample l:


$$O_{i,l}=\left\{ \begin{array}{l} \;1\;if\;taxon\;i\;present\;in\;sample\;l\\ 0\;otherwise\; \end{array}\right..$$

The statistic of interest is the difference in co-occurrence between the conditions for each pair of taxa, that is computed as


$$\Delta f_{i,j}^{o}=f_{i,j}^{cnd}-\;f_{i,j}^{ctr}.$$

The significance of $∆f_{i,j}^{o}$ is evaluated by means of two different models:

- a random co-occurrence model, which is obtained by repeatedly shuffling the elements of the occurrence matrices row-wise N times at random and each time calculating the $∆f_{i,j}$ to construct for each taxon pair a probability distribution of differential co-occurrence in the random co-occurrence scenario (only taxon frequency rates $f_{i,i}^{k}$ are conserved);
- a permutation co-occurrence model, which is obtained by repeatedly permuting the condition labels of the samples M times at random and each time calculating the $∆f_{i,j}$ to construct for each taxon pair a probability distribution of differential co-occurrence in the scenario of random grouping of samples (where the group sizes $n_{cnd}$ and $n_{ctr}$ are conserved).

The value $∆f_{i,j}^{o}$ is compared with the values $\mu_{\Delta f_{i,j}}\pm n{∙\sigma }_{\Delta f_{i,j}}$ (respectively above [+] and below [−]) to determine whether the observed differential co-occurrence is simply explained by chance given the occurrence frequencies of the taxon pair in each condition.

In parallel, the value $∆f_{i,j}^{o}$ is compared with the quantile values $Q\left(p\right)$ and $Q\left(1-p\right)$ of the distribution representing the permutation co-occurrence model to determine whether the observed differential co-occurrence is simply explained by clustering samples by chances given characteristic group sizes.

In the analyses performed here, we set a soft threshold of n=1 on the random model and a threshold of p=0.05 on the permutation model to weight more differences between conditions in the discovery process.

If $∆f_{i,j}^{o}$ differ more than $n{∙\sigma }_{\Delta f_{i,j}}$ from the value $\mu_{\Delta f_{i,j}}$ of the random model in any direction, then

- if $∆f_{i,j}^{o}$ is smaller than $Q\left(p\right)$, the co-occurrence between taxa i and j is under-represented in the condition samples compared to the control samples;
- if $∆f_{i,j}^{o}$ is larger than $Q\left(1-p\right)$, the co-occurrence between taxa i and j is over-represented in the condition samples compared to the control samples.

The significant differential co-occurrences identified can be regarded as proxies for ecological interactions between microbiota species that are strengthened or weakened when passing from one host condition to another. These can also be naturally represented as a colored network where the nodes represent taxa, and the links describe over- or under-represented co-occurrences in one condition versus the other according to the color.

Critically, our method requires that detected co-occurrences must satisfy statistical significance criteria from both the permutation null model and the random null model. This dual-test requirement reduces false discoveries and provides inherent robustness against any single test performing poorly on a particular data set, even in a discovery-oriented setting, where relaxed parameters are adopted to maximize sensitivity to potential ecological associations.

### Differential HOI analysis

The procedure to extract differential HOIs is analogous to the one described for the extraction of differential co-occurrences but considers the co-presence of a number of different microbiota species that equals the order of interaction to be investigated.

In the case of three-species interactions, the co-occurrence matrices are obtained by computing the frequencies at which triplets of taxa are co-present in the samples associated with the same host condition:


$$f_{i,j,t}^{k}=\frac{1}{n_{k}}\sum_{l\;\in \;condition\;k}O_{i,l}\cdot O_{j,l}\cdot O_{t,l}.$$

### Method computational complexity

The computational complexity of our differential co-occurrence analysis can be expressed in terms of the standard Big-O notation, where *n* represents the number of samples, *p* represents the number of taxa (after prevalence filtering), and *k* represents the number of reshufflings/permutations in the null random/permutation model.

The initial binarization step, converting relative abundance to presence-absence, operates over all *n* samples and *p* taxa, yielding *O*(*np*) complexity. The empirical co-occurrence computation stage calculates the *p* × *p* co-occurrence matrix for each condition (e.g., healthy versus disease state), requiring *O*(*p*^2^) operations total. These initial stages are negligible in comparison to the null model computations. The permutation null model shuffles sample labels between conditions *k* times while preserving the sample-taxon association structure. For each permutation, we recalculate the *p* × *p* co-occurrence matrices for both conditions, yielding *O*(*k* × *p*^2^) computational complexity. For each of *k* randomizations, the random null model shuffles the rows of the co-occurrence matrix to randomize which taxa co-occur while preserving the network degree distribution. This row shuffling and matrix reconstruction also requires *O*(*k* × *p*^2^) operations. Consequently, the dominant computational complexity is *O*(*k* × *p*^2^), where *k* represents the iterations in the null models. This complexity scales linearly with the number of iterations *k* and quadratically with the number of taxon *p*, but critically, it is independent of sample size *n*. This independence from sample size means our method scales favorably to large cohort studies.

On the other hand, HOI analysis faces a combinatorial challenge. Naively considering all possible triplets of taxa requires examining *C*(*p*,3) = *P*(*p* − 1)(*p* − 2)/6 combinations. However, in practice, the method restricts HOI testing to triplets that form triangles with a consistent sign in the differential co-occurrence network. That is, we only test triplets where all three pairwise associations were significant in the differential co-occurrence analysis and consistently over-represented in healthy versus disease or vice versa. For *t* equal to the number of consistent triangles (where *t* << *p*^3^), the complexity becomes *O*(*k* × *t*), resulting in the HOI analysis being computationally feasible and scalable.

### Statistical methods

To validate the method, microbiota-based clusters of patients obtained by clustering the abundance profiles of species selected by the differential co-occurrence analysis were tested for enrichment in the two conditions using the Freeman-Halton (two-tailed) extension of the Fisher exact probability test.

To account for the compositionality of microbiome data, taxon abundance profiles (counts) were transformed using the centered log-ratio (clr) transformation following imputation of zeros via Geometric Bayesian Multiplicative replacement method and then standardized across the patient population to obtain *z*-score values.

To hierarchically cluster patients, the Euclidean distance and the Ward linkage method were applied to patient abundance profiles (after clr-transformation and standardization) of differentially co-occurrent taxa.

### Phenotype analysis

We annotated differentially co-occurrent species by using the bacteria-archaea-traits-1.0.0 data set, which contains bacterial and archaeal phenotypic traits and environmental data synthesized from multiple phenotype databases (26).

Specifically, we used sets of continuous and categorical phenotypic traits condensed at the level of species and restricted by isolation type equal to “host” or “host_[ ]” and superkingdom type equal to “Bacteria,” to filter out data from archaea and bacteria not isolated from hosts, resulting in a subset of annotations from *N* = 2,717 bacterial species.

We retained and analyzed only the trait categories displaying less than 50% of missing values.

For categorical traits, we performed enrichment analysis of network blocks using Fisher’s exact test, while for quantitative traits (associated with integer or real-valued variables), we performed both Welch’s two-sample *t*-tests and *F*-tests between the set of species of the first block versus the set of species of the second block.

### Correlation network extraction

To construct relevance networks for the purpose of comparing them with the differential co-occurrence networks extracted by our method, we measured all pairwise Pearson’s correlation coefficient $C_{ij}$ between species i and j based on their clr-transformed abundance profile across samples, and we defined the correlation network as


$$a_{ij}=\left\{ \begin{array}{l} 1\;\;\;\;\;if\;|C_{ij}|\geq r\\ \;0\;\;\;\;otherwise \end{array}\right.,$$

where r is the minimum value of the correlation threshold for which $n_{r}$ links are extracted.

## RESULTS

### Case study: Pittsburgh melanoma cohort

Differential co-occurrence analysis was performed on the melanoma cohort (*n* = 63) to identify ecological network blocks of the gut microbiota that are characteristic of patients non-responding to anti-PD-1 immunotherapy. Prior to the analysis, LKTs showing a relative abundance value greater than 1% in less than 10% of the patients were assumed to have a less relevant functional impact on the gut ecology and were filtered out (Fig. S1).

We constructed the null models by performing N = M = 8,000 shufflings/permutations. A sensitivity analysis on these parameters confirmed that the number of significant co-occurrences already reached a plateau for N = M ≥ 2,000 (Fig. S2).

**Fig 2:**
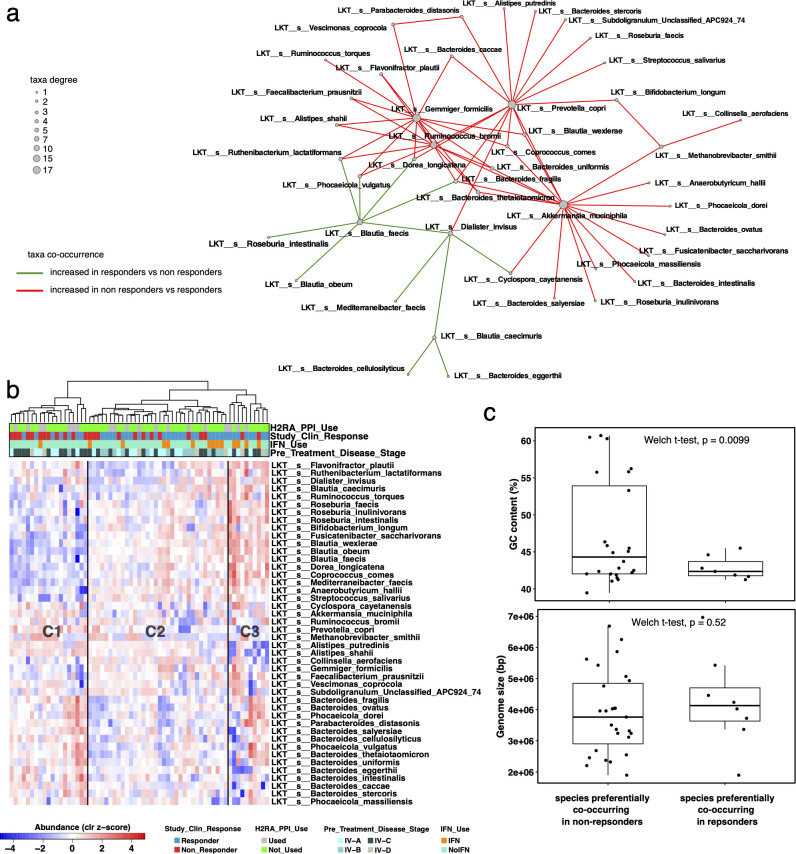
Pittsburgh melanoma cohort: (**a**) The microbiota ecological network modules at the level of last known taxa (LKT) extracted from baseline microbiome data of melanoma patients who either responded or did not respond to anti-PD-1 treatment by using the co-occurrence differential analysis; red/green links connect taxa that preferentially co-occur in non-responders/responders; in the plot, the size of each node (taxa) within the network is proportional to its network degree (number of incident links). (**b**) Hierarchically clustering the patients in the cohort (heatmap columns) based on the abundance profile of the bacterial species within the network (LKT, heatmap rows) identified three patient groups with significantly different rates of response to treatment, as highlighted with the metadata feature Study_Clin_Response in the covariate bar. (**c**) Comparison of genome size and genomic GC content of species preferentially co-occurring in responders versus species preferentially co-occurring in non-responders; the two-sample Welch *t*-test was used to assess differences in the average value of each feature between non-responder and responder patients.

Figure 2a shows the network of differential co-occurrence, consisting of 44 nodes and 68 interactions divided into two connected blocks. A first block of co-occurrences that are increased in responders versus non-responders (green links) revealed different species of the genus *Blautia* (*Blautia faecis*, *Blautia obeum*, and *Blautia caecimuris*). A second block, describing co-occurrences increased in non-responders compared to responders (red links), includes the species *Akkermansia muciniphila*, *Gemmiger formicilis*, *Prevotella copri*, and *Ruminococcus bromii* among its nodes with the highest number of interactions. No significantly HOIs were detected within this network.

When the normalized clr-transformed abundance of the species present as nodes in the network was used to cluster patients into three groups by hierarchical clustering (Fig. 2b), we could identify a group of 34 patients with a non-responder rate of 32.4% (C2), consistent with the 37% rate observed within the population overall, a second group of 19 patients (C1), with an increased rate of 63.2%, and a third group of 10 patients (C3) with only 20% of non-responders (Freeman-Halton *P* = 0.04).

To test that the co-occurrence network extracted did not encode trivial information, we repeated the clustering procedures starting from the nodes of a correlation network extracted by measuring all pairwise correlation coefficients between species abundance profiles and thresholding them to retain the same number of links (see Materials and Methods for details; Fig. S3a). The three clusters obtained in this way (Fig. S3b) did not show a significantly different response rate (Freeman-Halton *P* = 0.87).

To characterize the co-occurrence network at the phenotype level, we annotated the set of nodes in each network block separately. The list of annotated species specific to the first block (*n* = 7), specific to the second block (*n* = 31), and shared between blocks (*n* = 6) with related traits is reported in Tables S1 and S2.

The enrichment analysis of categorical traits on the blocks (Table S3) revealed that species preferentially co-occurring in non-responder patients (second block) are enriched by “no motility” (86%, Fisher *P* = 0.015) and “bacillus cell shape” phenotype (85%, Fisher *P* = 0.043). From a metabolic perspective, while in both blocks a significant over-representation (Fisher *P* < 0.05) of anaerobic species and an under-representation of aerobic species was detected, which reflected the common characteristic environment (human intestine), the second block also displayed a significant over-representation of obligate anaerobes (11%, Fisher *P* = 0.03) and under-representation of microaerophilic species (0%, Fisher *P* = 0.03).

Quantitative trait analysis (Table S4) revealed that, at the genomic level, those species preferentially co-occurring in responder patients (first block) have a significantly lower GC content compared to the species characteristic of non-responder ecological interactions (second block: mean first block 42.9%; mean second block 47.2%; Welch *t*-test *P* < 0.01) despite having comparable genome sizes (Fig. 2c).

The significance of these findings was confirmed when repeating the analysis after removing the species common to both blocks and using only the block-specific sets of species (Tables S5 and S6).

### Case study: comparative analysis of CRC cohorts

To demonstrate how differential co-occurrence can be used to compare data sets of different studies, we analyzed the CRC cohorts (Gupta *n* = 59, IEO *n* = 59 after raw sequencing data from one patient failed to be processed due to poor sequencing depth). Again, for each cohort, we filtered out LKTs with a relative abundance of at least 1% in less than 10% of the patients (Fig. S4 and S5), and we constructed the null models using N = M = 8000.

**Fig 3:**
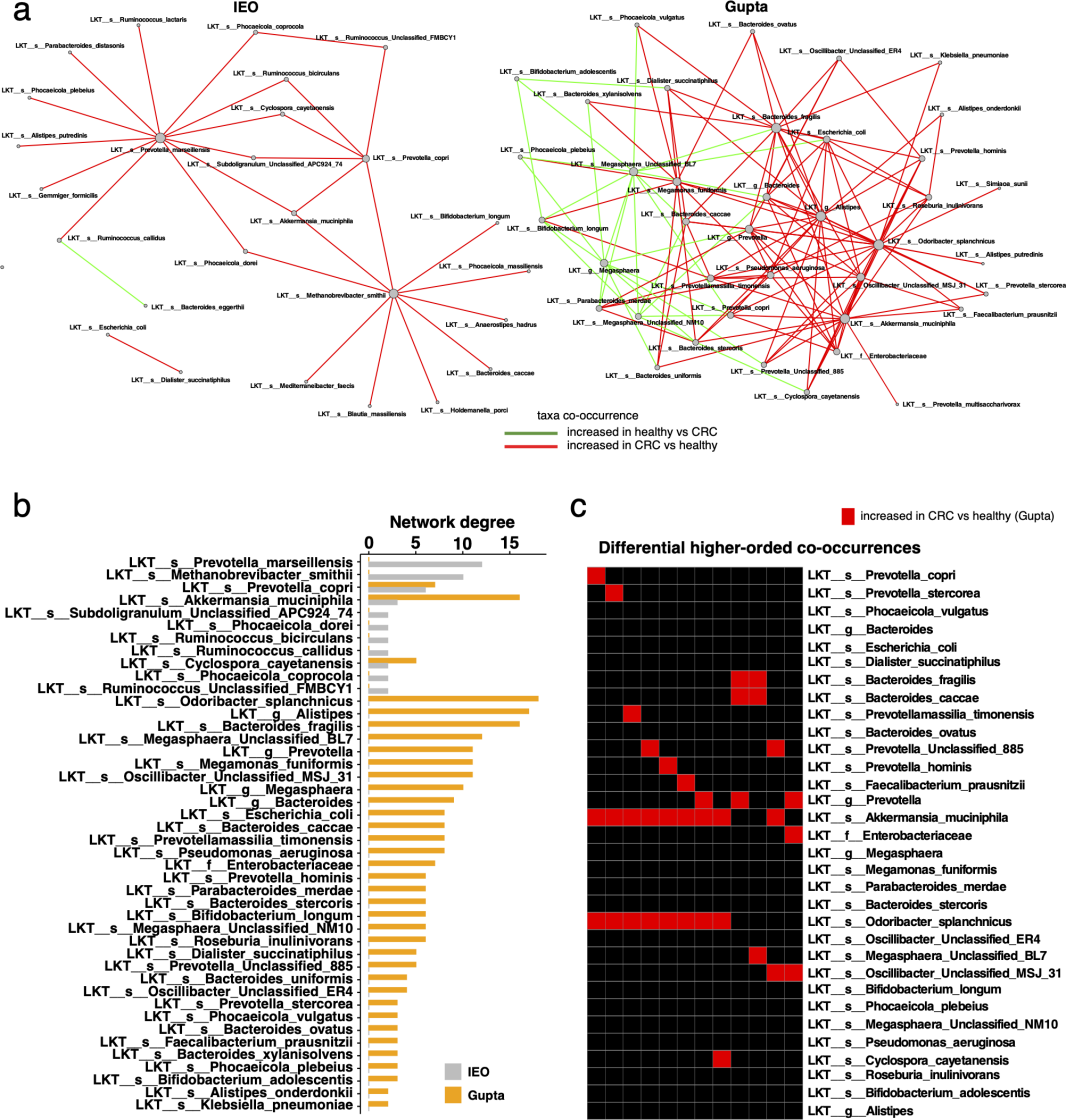
Comparative analysis of colorectal cancer (CRC) cohorts: (**a**) Microbiota ecological networks extracted from microbiome data of two different cohorts (IEO Italian left, Gupta Indian right) including both CRC patients and healthy subjects; red/green links connect taxa that preferentially co-occur in non-responders/responders; in the plot, the size of each node (taxa) within the network is proportional to its network degree (number of incident links). (**b**) Analysis of centrality of those species appearing in both ecological networks (IEO and Gupta), measured as network degree centrality (number of incident links to a node/species). (**c**) Higher-order interactions extracted from the Gupta CRC cohort microbiome data by means of differential co-occurrence analysis; each column of the heatmap shows a triplet of taxa (identified by the red boxes) that preferentially co-occur in CRC patients compared to healthy individuals.

Figure 3a shows the two differential co-occurrence networks extracted from the cohorts. Despite a comparable ratio of CRC cases over healthy (30/59 = 0.54 for Gupta versus 32/59 = 0.51 for IEO), the network extracted from the Indian cohort appears denser than the network from the Italian cohort, with 132 links between 39 nodes, with an average node degree equal to 6.8, compared to 30 links between 29 nodes and an average degree of 2.3 (IEO), reflecting a higher ecological complexity in the microbiota of non-Westernized populations.

Also, in this case, when the abundance of the species in the network was used to perform hierarchical clustering of samples regardless of patient response, the resulting clusters of patients encoded the relevant information with respect to patient conditions (Fig. S6 and S7).

Figure 3b summarizes the comparative analysis of the species between the cohorts based on their degree of centrality. In the IEO network (gray bars), the species with degree above average (high-centrality nodes) are all included within the sub-network of co-occurrences over-represented in the intestinal microbiome of CRC patients compared to healthy individuals. They include *Prevotella marseillensis* and *Methanobrevibacter smithii* (specific to the Italian cohort and not present in the Gupta network), *Prevotella copri* and *Akkermansia muciniphila* (also displaying high centrality in the Gupta network).

In the Gupta network (orange bars), 16 species had higher-than-average degree. Among them, *Odoribacter splanchnicus*, *Akkermansia muciniphila*, *Megamonas funiformis*, *Oscillibacter Unclassified_MSJ_31*, *Prevotellamassilia timonensis*, *Pseudomonas aeruginosa*, and two species having last known taxa of genus *Alistipes* (*LKT_g_Alistipes*) and family *Enterobacteriaceae* (*LKT_f_Enterobacteriaceae*), respectively, were exclusive of the block of co-occurrences characteristic within CRC. On the other hand, *Megasphaera_Unclassified_BL7* and *LKT_g_Megasphaera* were exclusive of the block of co-occurrences characteristic of a healthy microbiome, similar to *Megasphaera Unclassified NM10* and *Bifidobacterium adolescentis*, despite having a lower-than-average degree (6 and 3, respectively).

Three-node cliques in the networks were tested for the presence of differential HOIs. In the Gupta network, 12 cliques of the block of interactions characteristic of CRC microbiota resulted as significant for three-species co-presence (Fig. 3c). Interestingly, nine of the HOIs (75%) involve *Akkermansia muciniphila*, eight (67%) involve *Akkermansia muciniphila* and *Odoribacter splanchnicus* together, and nine (75%) involve at least one *Prevotella* species, suggesting that specific functional constraints accompany ecological HOIs.

Phenotypic analysis performed on the subset of species involved in HOIs (*n* = 7 species annotated; Tables S7 and S8) revealed an enrichment for gram negativity (all seven species, *P* = 0.017) and that most of the HOIs involve a combination of a coccobacillus (*Akkermansia muciniphila*), a fusiform (*Odoribacter splanchnicus*), and a bacillus (*Prevotella* species, *Faecalibacterium prausnitzii*), suggesting that heterogeneity in bacterial cell shape might play a role in determining higher-order co-occurrence of bacterial species.

## DISCUSSION

The analysis of the Pittsburgh melanoma cohort revealed that different *Blautia* species might mediate and facilitate the response to immune checkpoint inhibitors in melanoma patients. The documented ability of some *Blautia* species to produce bacteriocins and to biotransform bioactive substances (including flavonoids) could be explanatory factors for the observed centrality of this genus in the intestinal microbiota ecology of responding patients (27). Consistent with the hypothesis of a *Blautia* beneficial effect, the abundance of both *Blautia producta* and *Blautia wexlerae* was reported to associate with improved progression-free survival in the original publication of the cohort data (23).

Moreover, the phenotype analysis performed suggested that microbial species preferentially co-occurring in the microbiota of non-responder patients are characterized by a higher G + C content, on average, in the nucleotide composition of their genome compared to species preferentially co-occurring in patients who respond to anti-PD-1. No difference in average genome size was observed between the two sets of species. Alternative factors known to be associated with genomic base composition, including increased oxygen and nitrogen abundance or the ability to uptake foreign DNA from conjugation, transformation, and transduction, could play a role in the microbiota ecology of non-responder patients (28).

The applicability of the proposed method for the purpose of comparing microbiome data was demonstrated by performing the differential co-occurrence analysis on one Italian (IEO) and one Indian (Gupta) cohort including healthy individuals and CRC patients.

The two networks extracted highlighted differences in the intestinal microbiota ecology of CRC patients between the two geographically distinct populations.

In the microbiota of healthy Indian individuals, *Bifidobacterium adolescentis* (generally known for its benefits on health [29]), as well as species of the *Megasphaera* genus, appeared to have a pivotal role in those ecological interactions under-represented in the microbiota of Indian CRC patients.

While *Prevotella marseillensis* and *Methanobrevibacter smithii* were central species in the sub-network of interactions over-represented in CRC patients in the Italian cohort, *Odoribacter splanchnicus* appeared central to those in the Indian cohort. Moreover, two species, namely, *Prevotella copri* and *Akkermansia muciniphila*, appeared to play a central role in the CRC-altered microbiota ecology of both populations and, interestingly, also in melanoma patients non-responding to anti-PD-1 treatment.

Despite commonly being considered a next-generation probiotic for its metabolic functions and mucin-degrading capacity, *Akkermansia muciniphila* has been previously associated with increased abundance within CRC in both humans and mice (30).

Conflicting evidence exists on the association of *Prevotella copri* with human inflammatory disease states or on its beneficial effects on the host metabolism, and computational analyses attempting to explain the observed heterogeneity through functional diversity at the clade level were also inconclusive (31–33).

Our analysis explains these paradoxes at the level of microbe-microbe interactions and strongly indicates that the systematic co-presence of *Prevotella copri* and *Akkermansia muciniphila* is associated with cancer conditions in humans. However, it also suggests that *Prevotella copri* can associate with a healthy status in the Indian population when co-present with *Megasphaera* species, as evident in the Gupta network.

Additionally, the fact that higher-order ecological interactions detected in the Indian cohort ubiquitously involved *Akkermansia muciniphila*, *Odoribacter splanchnicus*, and species of the *Prevotella* genus and that those species also interact with *Megasphaera* species in healthy microbiota also suggests that the functional mechanism behind those interactions is not specific to *Prevotella copri* but rather shared by different *Prevotella* species.

Consistently, these findings came from the analysis of the Indian cohort, as *Prevotella* is a common gut-dominating genus in the non-Westernized populations with diets rich in plant-based food. In support of those findings, the high abundance of *Prevotella copri* was found to be associated with worse prognosis in the Pittsburgh melanoma cohort.

Interestingly, in the original publication from Gupta et al., where a standard differential abundance analysis was performed, *Akkermansia muciniphila* and *Odoribacter splanchnicus* were identified as CRC biomarkers, while *Prevotella copri* was identified univocally as a biomarker of healthy status.

By focusing on the statistics of co-occurrence, the method effectively questions the idea of probiotics in absolute terms, stressing the fundamental importance of ecological interaction in the microbiome and of their context-dependent nature.

Finally, HOIs were detected only in the Indian cohort and within the CRC-characteristic block of ecological interactions. As HOIs are defined here on the basis of co-occurrence, one hypothesis is that heterogeneity in bacterial cell shape reduces niche competition and facilitates the co-presence of opportunistic species with different morphologies in a disease condition (such as CRC), and this effect is more apparent in non-Westernized populations carrying a more diverse microbiota.

In the analyses presented, a default value of 0.01% was chosen for the detection threshold. This value was shown to be the optimal threshold for filtering sequencing artifacts in the task of mock community taxonomic classification across different state-of-the-art pipelines for shotgun metagenomic analysis, including JAMS (which was used here for processing raw metagenomic sequencing data) (34). Taxa effectively detected with a relative abundance below 0.01% can be expected to contribute negligibly to community-level function and metabolism, and this threshold has been used in studies of large data sets of human gut microbiota (35, 36).

Additionally, the core microbiota extraction step enforced in the analyses addressed the sampling zeros problem by effectively filtering out rare taxa whose detection can be compromised by sampling bias and is prone to false absence. We acknowledge the limitations of this approach, as the prevalence filtering criterion necessarily excludes rare pathogens and specialized symbionts that may be clinically or ecologically important. However, this exclusion is appropriate for co-occurrence analysis, which requires stable patterns of presence and absence across sufficient samples to reliably estimate associations.

### Conclusion

Recent evidence demonstrates that presence/absence associations in microbiome data reveal distinct ecological patterns that cannot be captured by abundance-based methods (37, 38).

Here, we have introduced a method, named differential co-occurrence analysis, that can be generally applied to extract networks from occurrence data sets with group labels by evaluating the preferential co-occurrences of the features in one group compared to another.

The method is specifically designed to extract ecological modules from clinical microbiome data, whose edges encode the preferential co-occurrence of species in the microbiota of individuals in specific clinical conditions (for example, cancer or negative treatment response) versus control individuals, and vice versa. Moreover, the method can be used to study co-occurrences of orders higher than pairwise that, in turn, can proxy higher-order ecological interactions of bacteria in the intestinal microbiota.

To benchmark the method, three cancer microbiome data sets with a comparable ratio of cases-over-controls were processed using the JAMS pipeline and analyzed. The results reported here demonstrate that our network-based approach can extract relevant information that cannot be retrieved otherwise by using standard differential abundance analysis. Furthermore, when used in combination with data from functional annotation databases, new functional insights into the ecology of the microbiota in health and disease can be provided.

Our method enforces presence/absence statistics as a robust source of information for detecting group effects with respect to noise and confounders affecting the statistics of abundance values in clinical microbiome data. However, edges in the differential co-occurrence networks remain associational and may still reflect shared host or environmental factors rather than direct microbe-microbe interactions.

In presenting the method, we focused on its application to the analysis of clinical microbiome data, for which it was designed; nevertheless, the differential co-occurrence analysis methodology can be applied to any ecology data set and used in other research fields where occurrence data are studied.

## Data Availability

The data sets analyzed during the current study are available in the NCBI database: Gupta cohort, BioProject accession numbers PRJNA531273 and PRJNA397112; IEO cohort, accession number PRJNA447983; Pittsburgh cohort, BioProject accession number PRJNA762360. The JAMS software for processing raw metagenomics data is available at the following repository: https://github.com/johnmcculloch/JAMS_BW. The R implementation of the differential co-occurrence analysis method (package difecomod) is available on github at the following repository https://github.com/CMONLab/difecomod. The package is installable via devtools::install_github ("CMONLab/difecomod") and includes a step-by-step tutorial demonstrating the complete analytical workflow.

## SUPPLEMENTAL MATERIAL

Supplemental material msystems.00284-26-s0001.pdf Figures S1–S7 and supplemental table legends.

Table S1 msystems.00284-26-s0002.xlsx Categorical phenotypic annotations from the bacteria-archaea-traits-1.0.0 data set.

Table S2 msystems.00284-26-s0003.xlsx Quantitative phenotypic annotations from the bacteria-archaea-traits-1.0.0 data set.

Table S3 msystems.00284-26-s0004.xlsx Results of the enrichment analysis for categorical phenotypic annotations from the bacteria-archaea-traits-1.0.0 data set.

Table S4 msystems.00284-26-s0005.xlsx Results of the differential analysis of quantitative phenotypic traits from the bacteria-archaea-traits-1.0.0 data set.

Table S5 msystems.00284-26-s0006.xlsx Results of the enrichment analysis for categorical phenotypic annotations from the bacteria-archaea-traits-1.0.0 data set when considering only block-specific sets of species.

Table S6 msystems.00284-26-s0007.xlsx Results of the differential analysis of quantitative phenotypic traits from the bacteria-archaea-traits-1.0.0 data set when considering only block-specific sets of species.

Table S7 msystems.00284-26-s0008.xlsx Categorical phenotypic annotations for the species involved in HOI.

Table S8 msystems.00284-26-s0009.xlsx Results of the enrichment analysis for categorical phenotypic annotations from the bacteria-archaea-traits-1.0.0 data set of the set of species involved in HOI.

Data S1 msystems.00284-26-s0010.xlsx Metadata associated with the samples analyzed in the study.

ASM does not own the copyrights to Supplemental Material that may be linked to, or accessed through, an article. The authors have granted ASM a non-exclusive, world-wide license to publish the Supplemental Material files. Please contact the corresponding author directly for reuse.
