# Supplementary material for: Differential co-occurrence analysis: a method to extract ecological modules from clinical microbiome data
Source: mSystems. 2026 Jun 9;11(7):e00284-26. doi: 10.1128/msystems.00284-26 (PMC13387006; doi:10.1128/msystems.00284-26)
Supplement: Supplemental material — Figures S1–S7 and supplemental table legends. [file msystems.00284-26-s0001.pdf]

Supplementary Material: Differential Co-occurrence Analysis: a Method to Extract Ecological Modules from Clinical Microbiome Data

J. Iacovacci[1,*], N. Cannon[2], J. A. McCulloch[2], T. Rancati[1,$], G. Trinchieri[2,$]

*Correspondence: jacopo.iacovacci@istitutotumori.mi.it
[$] Co-last authors

Affiliations:

[1] Data Science Unit, Fondazione IRCCS Istituto Nazionale dei Tumori di Milano, Milan, Italy.

[2] Laboratory of Integrative Cancer Immunology, National Cancer Institute Center for Cancer Research, Bethesda, Maryland, USA.

Supplementary Figures

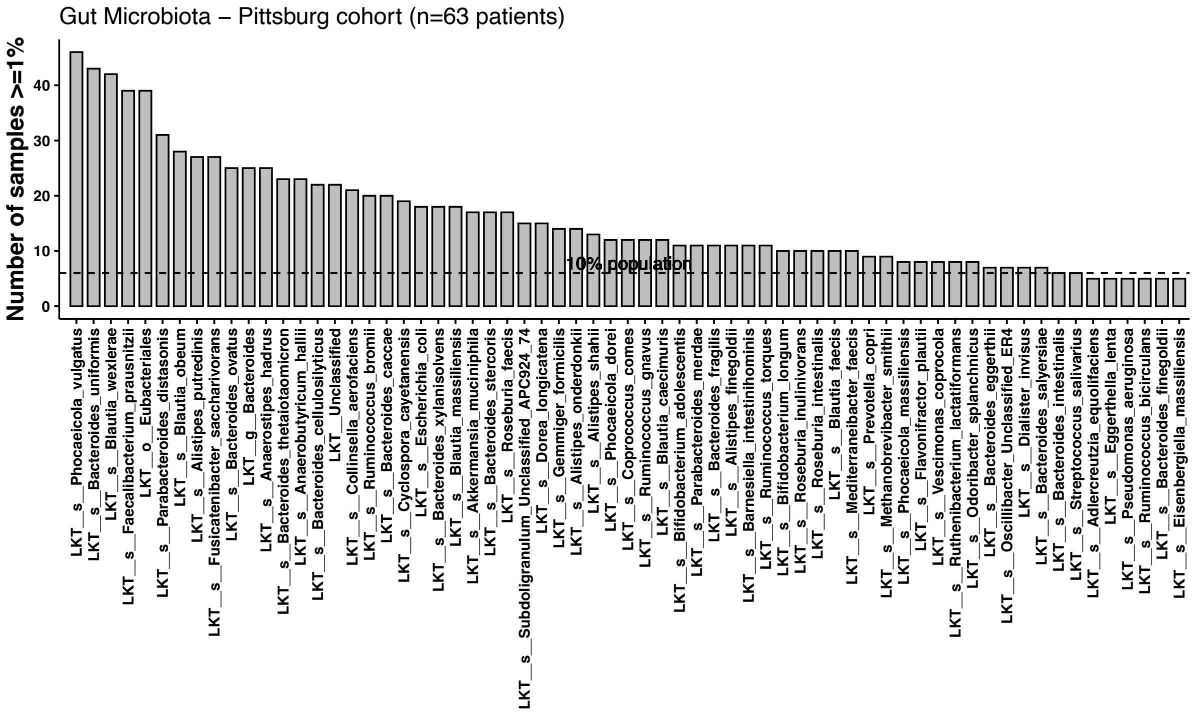

Supplementary Figure S1. Bar plot showing the number of times intestinal microbiota species (LKT: Last Known Taxa) were detected with a relative abundance ≥1% across patients in the Pittsburgh cohort.

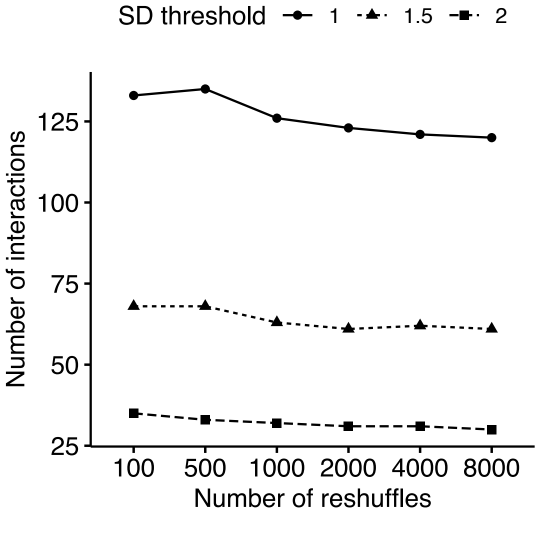

Supplementary Figure S2. The number of network interactions detected by the co-occurrence differential analysis in function of the number of reshuffles of the rows of the presence/absence matrices for three different significance threshold values expressed in units of of standard deviation (SD) of the random null model distribution.

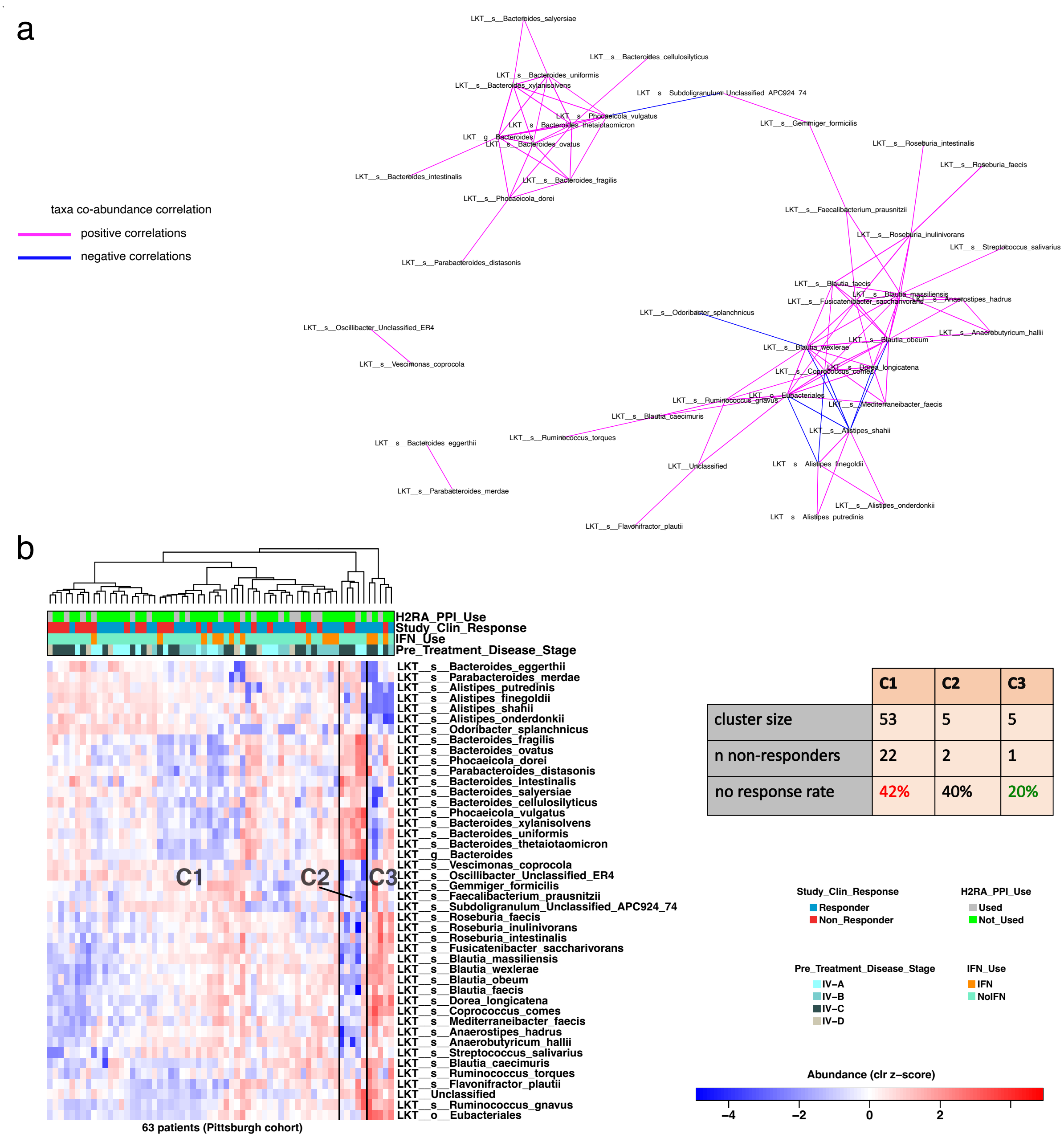

	C1	C2	C3
cluster size	53	5	5
n non-responders	22	2	1
no response rate	42%	40%	20%

Supplementary Figure S3: (a) The relevance network between species (LKT: Last Known Taxa) extracted from baseline microbiome data of melanoma patients by retaining the highest coefficients of correlations (purple) or anticorrelations (blue) in abundance variations across patients; correlations were measured with Pearson's correlation coefficient and filtered at the minimum threshold to connect n=44 species (i.e., same number of species as in Figure 1b). (b) Hierarchically clustering the patients in the cohort (heatmap columns) based on the abundance profile of the bacterial species in the relevance network (LKT, heatmap rows) identified three clusters with no significantly different rates of response to treatment (Study_Clin_Response in the covariate bar, Freeman-Halton p ≥0.01).

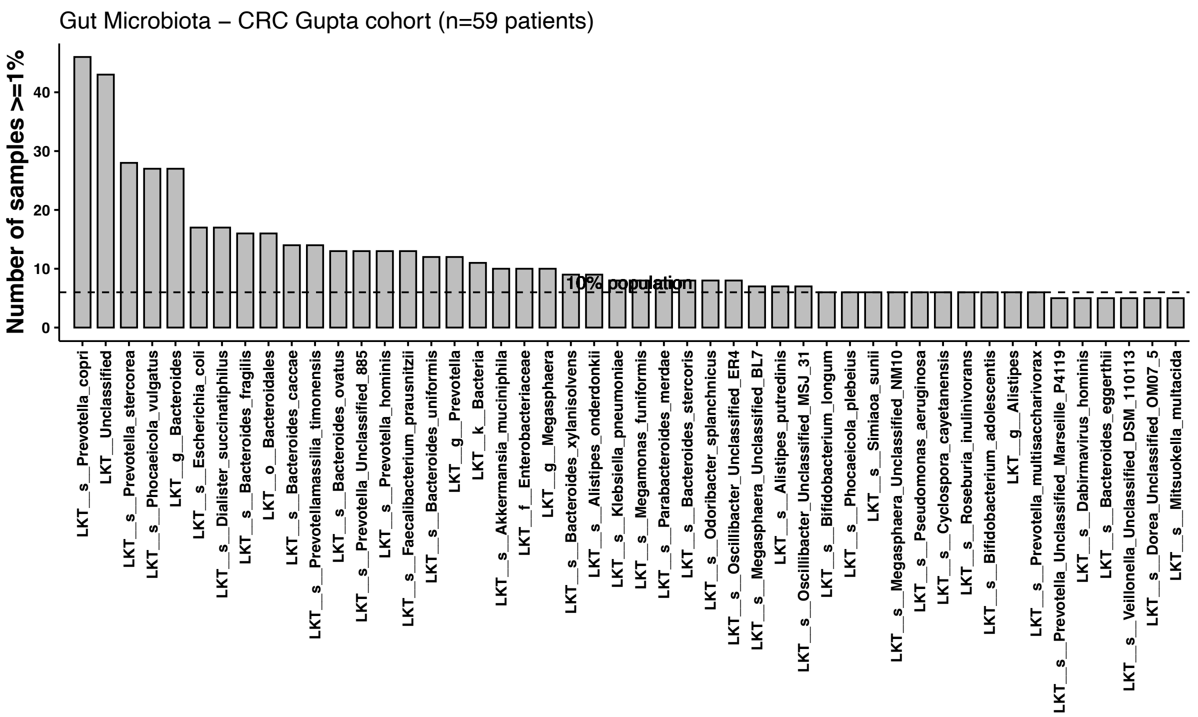

Supplementary Figure S4. Bar plot showing the number of times intestinal microbiota species (LKT: Last Known Taxa) were detected with a relative abundance ≥1% across patients in the Gupta cohort.

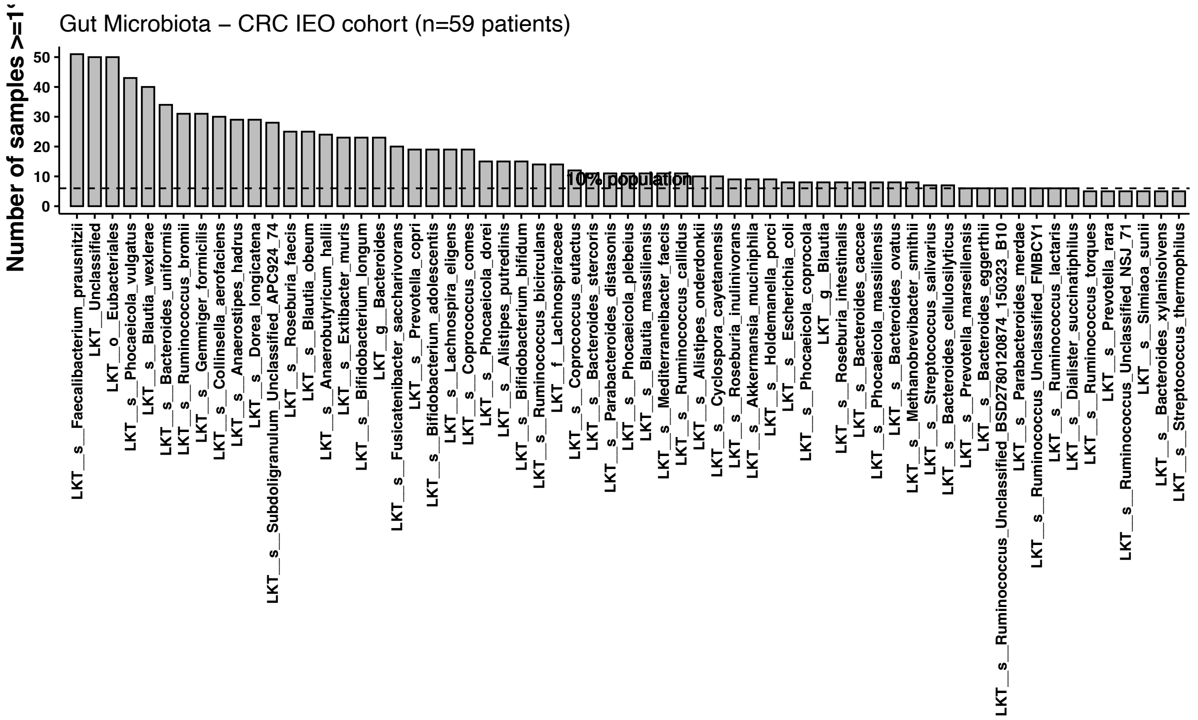

Supplementary Figure S5. Bar plot showing the number of times intestinal microbiota species (LKT: Last Known Taxa) were detected with a relative abundance ≥1% across patients in the IEO cohort.

Gupta cohort

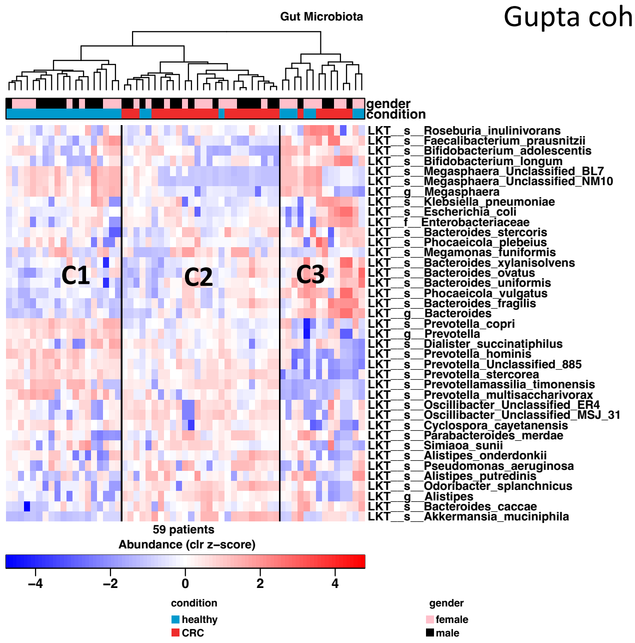

	C1	C2	C3
n cluster	19	26	14
n CRC	0	23	7
rate	0%	88.5%	50%

Freeman-Halton $p < 0.01$

Supplementary Figure S6. Hierarchically clustering the individuals in the Gupta cohort (heatmap columns) based on the abundance profile of the bacterial species within the Gupta network of Figure 3a (LKT, heatmap rows) identified three clusters with different incidences of individuals affected by colorectal cancer (CRC) as reported in the table on the right side and highlighted with the metadata feature condition in the covariate bar.

IEO cohort

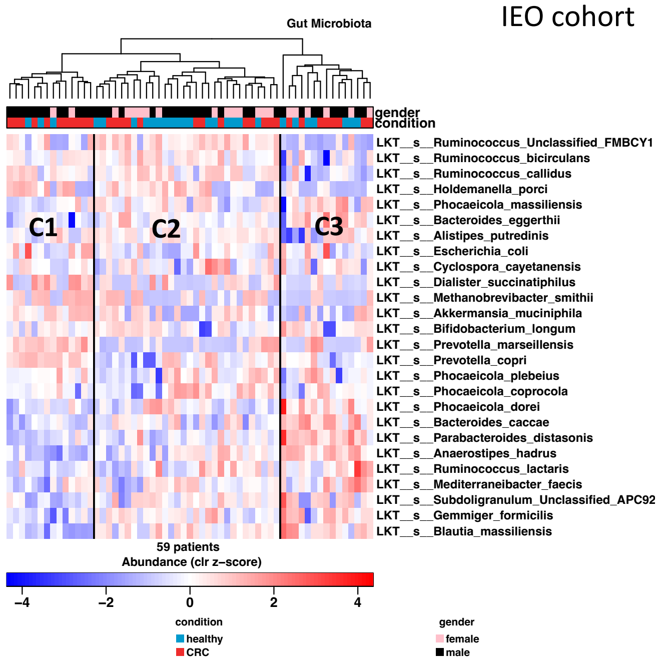

	C1	C2	C3
n cluster	14	30	15
n CRC	11	13	8
rate	78.6%	43.3%	53%

Freeman-Halton $p = 0.1$

Supplementary Figure S7. Hierarchically clustering the individuals in the IEO cohort (heatmap columns) based on the abundance profile of the bacterial species within the IEO network of Figure 3a (LKT, heatmap rows) identified three clusters with different incidences of individuals affected by colorectal cancer (CRC) as reported in the table on the right side and highlighted with the metadata feature condition in the covariate bar.

Supplementary Table Legends

Supplementary Table S1. List of categorical phenotypic annotations from the *bacteria-archaea-traits-1.0.0* data set for the species appearing uniquely in the ecological network module of responder patients (“block.or” in column “module”), or uniquely in the module of non-responder patients (“block.ur” in column “module”), or shared between modules (“block.int” in column “module”) as resulting from the analysis of the Pittsburgh cohort (see Figure 2a).

Supplementary Table S2. List of quantitative phenotypic annotations from the *bacteria-archaea-traits-1.0.0* data set for the species appearing uniquely in the ecological network module of responder patients (“block.or” in column “module”), or uniquely in the module of non-responder patients (“block.ur” in column “module”), or shared between modules (“block.int” in column “module”) as resulting from the analysis of the Pittsburgh cohort (see Figure 2a).

Supplementary Table S3: Results of the enrichment analysis (Fisher’s exact test) for categorical phenotypic annotations from the *bacteria-archaea-traits-1.0.0* data set (columns trait and class combined) of the ecological network modules (column module) extracted from the Pittsburgh data set.

Supplementary Table S4: Results of the differential analysis (Welch t-test, F-test) of quantitative phenotypic traits from the *bacteria-archaea-traits-1.0.0* data set (column trait) between the ecological network modules of responder versus non responder patients extracted from the Pittsburgh data set.

Supplementary Table S5: Results of the enrichment analysis (Fisher’s exact test) for categorical phenotypic annotations from the *bacteria-archaea-traits-1.0.0* data set (columns trait and class combined) of the ecological network modules (column module) extracted from the Pittsburgh data set when considering only block-specific sets of species.

Supplementary Table S6: Results of the differential analysis (Welch t-test, F-test) of quantitative phenotypic traits from the *bacteria-archaea-traits-1.0.0* data set (column trait) between the ecological network modules of responder versus non responder patients extracted from the Pittsburgh data set when considering only block-specific sets of species.

Supplementary Table S7: List of categorical phenotypic annotations for the species involved in higher-order interactions (HOI) as resulting from the analysis of the Gupta data set.

Supplementary Table S8: Results of the enrichment analysis (Fisher’s exact test) for categorical phenotypic annotations from the *bacteria-archaea-traits-1.0.0* data set (columns trait and class combined) of the set of species involved in higher-order interactions (HOI) as resulting from the analysis of the Gupta data set.
